# Early Detection and Management of Cognitive Impairment in Parkinson's Disease: A Predictive Model Approach

**DOI:** 10.1002/brb3.70423

**Published:** 2025-03-13

**Authors:** Li Li, Shan Tang, Bin Hao, Xiaoqin Gao, Haiyan Liu, Bo Wang, Hui Qi

**Affiliations:** ^1^ Department of Neurology First Hospital of Shanxi Medical University Taiyuan Shanxi People's Republic of China; ^2^ Department of Nursing First Hospital of Shanxi Medical University Taiyuan Shanxi People's Republic of China; ^3^ Shanxi Key Laboratory of Otorhinolaryngology‐Head and Neck Cancer First Hospital of Shanxi Medical University Taiyuan Shanxi People's Republic of China

**Keywords:** cognitive impairment, hematological markers, Parkinson's disease, prediction model, risk factors

## Abstract

**Objective:**

The study aims to identify risk factors associated with cognitive impairment in Parkinson's disease and to develop a predictive model to facilitate early clinical detection, diagnosis, and management, thereby enhancing patient prognosis and quality of life.

**Methods:**

A total of 351 PD patients were enrolled from the Department of Neurology at the First Hospital of Shanxi Medical University between January 2022 and December 2023. Cognitive function was evaluated using the Montreal Cognitive Assessment (MoCA) scale, and patients were subsequently categorized into cognitively normal (PD‐NC) and cognitively impaired (PD‐CI) groups. A logistic regression analysis was conducted to identify risk factors, and a predictive model was constructed and validated.

**Results:**

Among the 351 patients with PD, 188 cases were in the PD⁃NC group and 163 cases were in the PD⁃CI group, with an incidence of cognitive impairment of 46.4%. Logistic regression analysis indicated that H–Y classification, HAMA score, homocysteine, uric acid, and folic acid were significant predictors and were incorporated into the regression equation. The constructed prediction model had an area under the receiver operating characteristic curve of 0.738.

**Conclusions:**

The cognitive function of PD patients is influenced by H–Y classification, HAMA score, homocysteine, uric acid, and folic acid. The constructed prediction model demonstrates good discrimination and calibration, providing a reference basis for early clinical identification and intervention of cognitive impairment in Parkinson's disease patients.

## Abstract 2

This study identified H–Y classification, HAMA score, homocysteine, folic acid, and uric acid as independent predictors of PD‐CI and constructed the nomogram model to optimize early‐stage PD‐CI detection.

## Graphical abstract

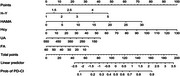

## 1 Introduction

Parkinson's disease (PD), the second most prevalent neurodegenerative disorder after Alzheimer's disease, is currently the neurological disease with the fastest increasing prevalence and disability worldwide (Feigin et al. 2021; Phongpreecha et al. 2020), affecting 2%–3% of individuals over the age of 65 years (Aarsland et al. 2021a). The main symptoms of PD are progressive motor disorders, including resting tremor, bradykinesia, myotonia, and postural balance disorders (Armstrong and Okun 2020), in addition to various non‐motor symptoms that may accompany the condition. Cognitive impairment, a prevalent non‐motor symptom (Marsili et al. 2018), is characterized by mental slowing, impaired abstract thinking, and difficulty in reasoning, affecting sensory perception, cognition, mood, motivation, autonomic function, and sleep quality. Cognitive impairment may manifest at various phases of PD (Aarsland et al. 2001), significantly impacting patients' social function and quality of life while exacerbating caregiver load and healthcare expenses (Bloem et al. 2021; Ulugerger Avci et al. 2023; Zhang et al. 2020).

There are currently no treatments to alter the disease process and no reliable early diagnostic biomarkers. Clinical observations indicate that certain people with PD have cognitive impairment at the time of diagnosis, implying that cognitive deficits may predate the onset of motor symptoms in these individuals (Liu et al. 2015). Consequently, recognizing early indicators of cognitive impairment risk in patients is critically important for mitigating or potentially correcting cognitive decline in individuals with PD. Research indicates that the Hoehn–Yahr (H–Y) classification reflects the progression of motor symptoms in PD. Patients at Stage 2 or higher show a 15% reduction in the thickness of the prefrontal cortex, which directly relates to a decline in executive function (Sharafkhah et al. 2024). Anxiety activates the hypothalamic–pituitary–adrenal (HPA) axis, resulting in a 30% increase in cortisol levels, which speeds up the apoptosis of hippocampal neurons. In a depressed state, the functional connectivity between the prefrontal cortex and the limbic system weakens, and dopaminergic projections are abnormal, resulting in impaired working memory (Russo and Nestler 2013). Studies show that early PD patients with comorbid anxiety and depression have a Montreal Cognitive Assessment (MoCA) score that is 2.8 points lower than those with just PD, and the rate of cognitive decline accelerates by 40% each year (Forbes et al. 2021). Elevated homocysteine (Hcy) impairs cognition through oxidative stress, and a deficiency in folate/B12 makes it even more toxic (An et al. 2019). The antioxidant effect of uric acid (UA) may help delay neurodegeneration, and low levels increase cognitive risk by 17% (Alrouji et al. 2024). Glycated hemoglobin levels over 6.5% worsen neuroinflammation through the AGEs‐RAGE axis (Wang et al. 2012). Lower ceruloplasmin levels lead to iron buildup in the brain, causing executive function impairment (W. Yu et al. 2019). Using combined multi‐indicator testing helps identify cognitive impairment in PD early. The aim of this study was to find and validate the influencing factors significantly associated with the risk of cognitive impairment in PD patients through multifactorial analysis, to construct a prediction model, and provide a scientific basis for early intervention.

## 2 Objects and Methods

### 2.1 Study Subjects

This retrospective study collected and collated the medical records of 351 patients with PD hospitalized in the Department of Neurology from January 2022 to December 2023 through the medical big data platform of the First Hospital of Shanxi Medical University in October 2024. Inclusion criteria: confirmed diagnosis of PD according to the Movement Disorder Society Parkinson's disease diagnostic criteria, and all patients were clinically diagnosed or diagnosed with probable idiopathic PD. Exclusion criteria: a history of other serious or unstable medical conditions interfering with the assessment of cognitive function; active epilepsy, aphasia, and impaired consciousness; a history of acute cerebrovascular disease in the past 3 months; a history of intracranial surgical treatments; a family‐related history of the disease; the presence of other primary etiologies of cognitive impairment; the presence of other symptoms associated with PD that can significantly affect cognitive function tests. This retrospective study was approved by the Ethics Committee of the First Hospital of Shanxi Medical University (Ethics number: NO.KYLL‐2024‐260) and was conducted following the 1964 Helsinki Declaration and its later amendments or comparable ethical standards. Informed consent was waived by our Institutional Review Board because of the retrospective nature of our study. This study was conducted in compliance with data protection regulations. The use of personal data was approved by the First Hospital of Shanxi Medical University Ethics Committee, ensuring that all data was anonymized and securely stored.

### 2.2 Data Collection

The general information of the patients was collected, including gender, age, and H–Y classification; H–Y classification was categorized as 0–5 based on motor severity. The scale information included the MoCA Scale, Hamilton Anxiety Scale (HAMA), and Hamilton Depression Scale (HAMD). Cognitive function was assessed using MoCA, a validated tool for PD‐CI screening (Nasreddine et al. 2005). A cutoff score of < 26 (sensitivity: 90%, specificity: 87%) was applied to define cognitive impairment, consistent with PD‐MCI Level I criteria (Litvan et al. 2012). Anxiety and depression severity were evaluated using HAMA and HAMD, both standardized instruments with established validity in PD populations. A cutoff score of ≥ 14 was applied to define anxiety and depression. The auxiliary examinations included nigrostriatal ultrasound, electroencephalogram, and the laboratory‐associated examinations comprised Hcy (normal value: 6–15µmol/L), folic acid (normal value: > 10nmol/L), vitamin B12 (normal value: 133–675pmol/L), UA (normal value: 210–440µmol/L), glycosylated hemoglobin (normal value: 4.8%–5.9%), ceruloplasmin (normal value: 0.2–0.6g/L), d‐dimer (normal value: 0–0.55mg/L), total cholesterol (normal value: 3.25–5.18mmol/L). Data on medication types (e.g., levodopa, dopamine agonists) and duration of use were not systematically recorded in the retrospective dataset. Similarly, years since diagnosis were unavailable for a subset of patients due to incomplete records, which precluded inclusion in analyses.

### 2.3 Statistical Methods

Data analysis and construction of risk prediction models were performed using SPSS 29.0 and R 4.4.0. Quantitative data conforming to normal distribution were expressed as mean ± standard deviation (*x* ± *s*), an independent samples *t*‐test was used for two‐group comparison, and analysis of variance (ANOVA) was used for multiple comparisons; quantitative data that did not conform to a normal distribution were expressed as median (interquartile spacing) [M(IQR)], and a rank‐sum test was used for two‐group comparisons; qualitative data were expressed as the number of cases and percentage (%), and *χ* ^2^ test was used for two‐group or multiple‐group comparison. The statistically significant variables in the univariate analysis were included in the multivariate logistic regression analysis to screen the independent risk factors associated with the occurrence of PD‐CI, and the screened independent risk factors were included as variables in the R 4.4.0 software, and the nomogram risk prediction model was plotted using rms. The area under the receiver operating characteristic curve (ROC), calibration curve, and clinical decision curve (DCA) was plotted by pROC, RMDA, and ggplot2 to assess the differentiation, calibration, and clinical application value of the prediction model. A two‐sided test was used in this study, and *p *< 0.05 was considered statistically significant.

## 3 Results

### 3.1 Univariate Analysis of Risk Factors for Cognitive Impairment in PD Patients

Based on the MoCA score, 351 patients were categorized into a cognitively normal group (PD‐NC, ≥ 26 points) and a cognitive impairment group (PD‐CI, < 26 points), comprising 188 patients in the PD‐NC group and 163 patients in the PD‐CI group, and the incidence of cognitive impairment was 46.4%. Univariate analysis revealed statistically significant differences in age, H–Y classification, HAMA score, HAMD score, Hcy, folic acid, vitamin B12, UA, and d‐dimer between the two groups (*p *< 0.05). (Table 1)

**TABLE 1:** Univariate analysis of risk factors for cognitive impairment in PD patients.

Characteristic		PD‐NC (*n* = 188)	PD‐CI (*n* = 163)	*χ* ^2^, *t*, *Z*	*p*‐value
Gender	Male	85	74	*χ* ^2^ = 0.001	0.972
	Female	103	89		
Age		64.17 ± 9.30	66.99 ± 7.37	*t* = −3.118	0.002
H–Y classification		2.03 ± 0.74	2.38 ± 0.78	*t* = −4.341	< 0.001
HAMA		9.31 ± 5.87	12.89 ± 5.77	*t* = −5.738	< 0.001
HAMD		9.50 ± 6.63	13.44 ± 6.56	*t* = −5.573	< 0.001
Nigrostriatal ultrasound	Normal	141	120	*χ* ^2^ = 0.087	0.768
	Abnormal	47	43		
Electroencephalogram	Normal	134	107	*χ* ^2^ = 1.287	0.257
	Abnormal	54	56		
Homocysteine		16.36 ± 7.36	20.64 ± 16.41	*Z* = −3.218	0.001
Folic acid		22.63 ± 12.69	19.88 ± 11.47	*Z* = 2.112	0.035
Vitamin B12		209.28 ± 111.18	183.35 ± 103.24	*Z* = 2.252	0.025
Uric acid		294.42 ± 77.68	268.25 ± 74.42	*Z* = 3.209	0.001
Glycosylated hemoglobin		5.76 ± 0.75	5.84 ± 0.54	*Z* = −1.214	0.113
Ceruloplasmin		0.24 ± 0.05	0.24 ± 0.04	*Z* = −0.350	0.727
d‐dimer		0.56 ± 0.74	0.75 ± 0.74	*Z* = −2.325	0.021
Total cholesterol		4.30 ± 0.97	4.17 ± 1.12	*Z* = 1.221	0.223

### 3.2 Multifactorial Analysis of Risk Factors for Cognitive Impairment in PD Patients

In the univariate analysis, statistically significant factors (age, H–Y classification, HAMA score, HAMD score, Hcy, UA, folic acid, vitamin B12, and d‐dimer) were designated as independent variables, while the categorization of patients' cognitive function served as the dependent variable (PD‐NC = 0, PD‐CI = 1) for the logistic regression analysis, utilizing the original values of the independent variables. The results indicated that H–Y classification, HAMA score, Hcy, folic acid, and UA were incorporated into the regression equation and identified as independent risk factors for cognitive impairment in PD patients. (Table 2)

**TABLE 2:** Logistic regression analysis affecting cognitive function in PD patients.

Variant	*β*	SE	Wald *χ* ^2^	*p*	OR	95% CI
Age	0.023	0.015	2.313	0.128	1.023	[0.993, 1.055]
H–Y classification	0.358	0.168	4.542	0.033	1.430	[1.029, 1.988]
HAMA	0.068	0.033	4.182	0.041	1.070	[1.003, 1.143]
HAMD	0.033	0.029	1.352	0.245	1.034	[0.977, 1.094]
Homocysteine	0.025	0.013	4.030	0.045	1.026	[1.001, 1.052]
Folic acid	−0.020	0.010	3.936	0.047	0.980	[0.960, 1.000]
Vitamin B12	−0.002	0.001	1.827	0.177	0.998	[0.996, 1.001]
Uric acid	−0.003	0.002	4.410	0.036	0.997	[0.994, 1.000]
d‐dimer	0.214	0.164	1.706	0.191	1.239	[0.898, 1.707]

### 3.3 Construction of Risk Prediction Model for Cognitive Impairment in PD Patients

**FIGURE 1:**
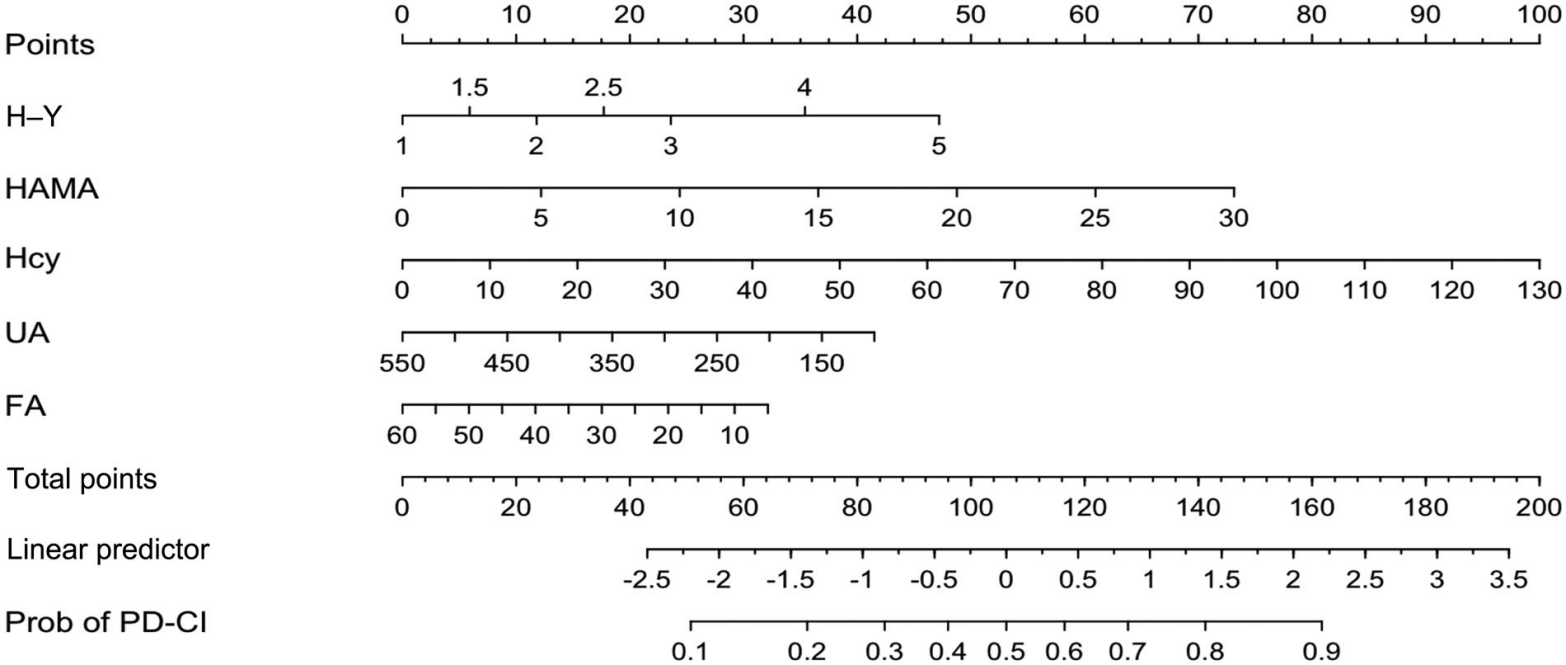
Risk prediction model of cognitive impairment in PD patients.

The risk prediction model of cognitive impairment in PD patients was constructed by including the five independent variables with *p *< 0.05 in the multifactor logistic regression analysis in the nomogram model. (Figure 1). Nomograms visually quantify individual risk by integrating multiple predictors, enhancing clinical utility.

### 3.4 Validation of Risk Prediction Model for Cognitive Impairment in PD Patients

**FIGURE 2:**
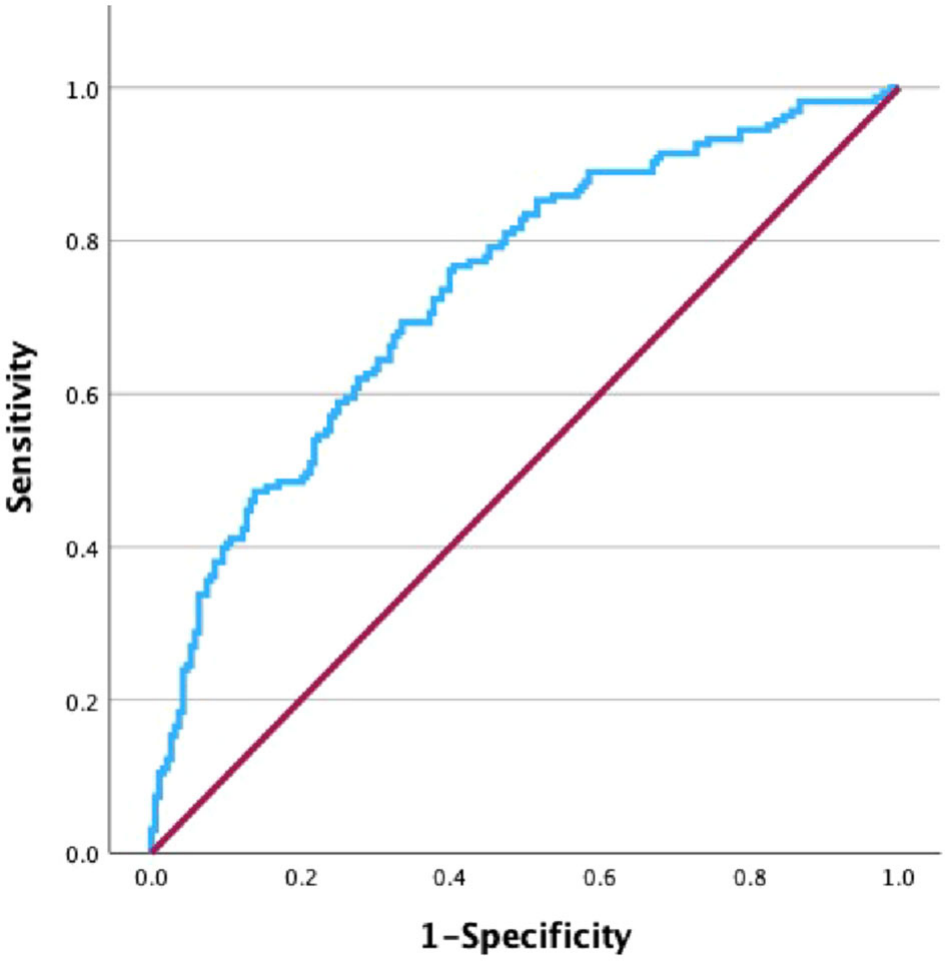
ROC curves of the cognitive impairment prediction model in PD patients.

**FIGURE 3:**
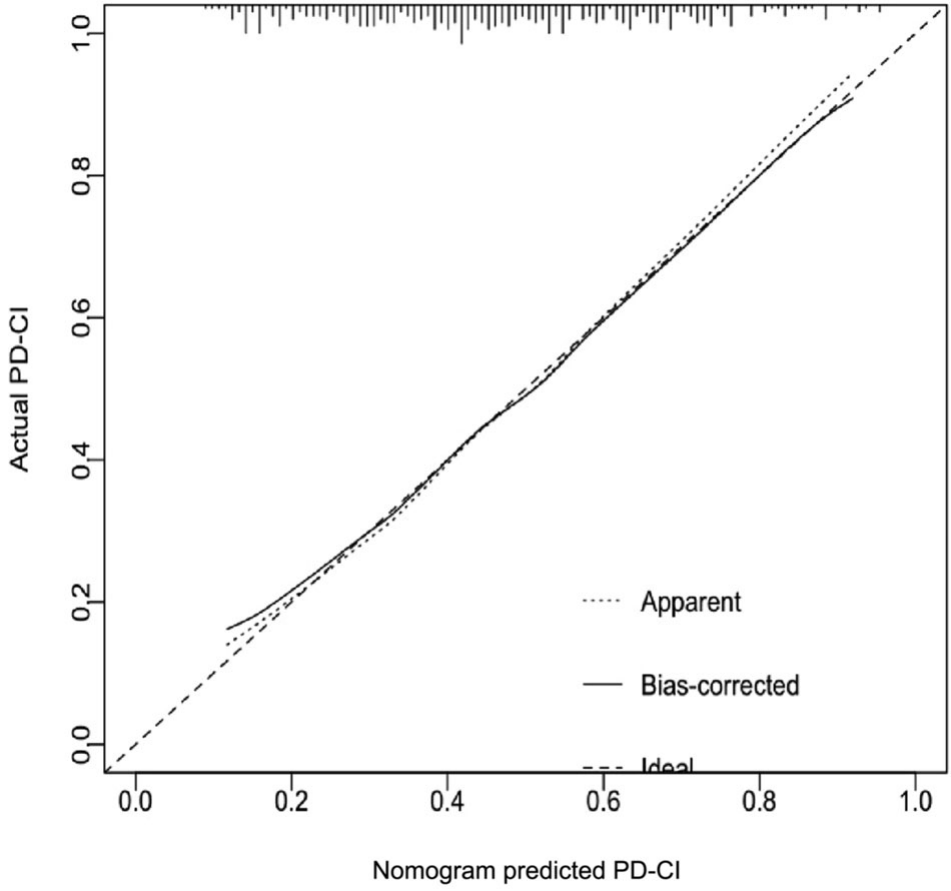
Calibration curve of the cognitive impairment prediction model in PD patients.

**FIGURE 4:**
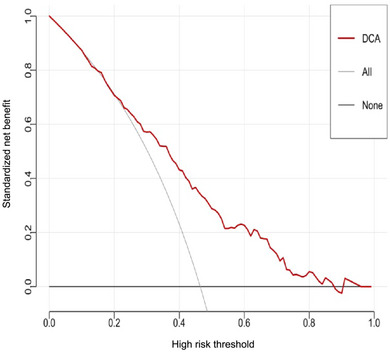
Clinical decision curve of cognitive impairment prediction model in PD patients.

ROC‐AUC reflects the model's ability to distinguish PD‐CI from PD‐NC. The Hosmer–Lemeshow test (via calibration curve) confirms agreement between predicted and observed risks. Decision curve analysis demonstrates net benefit across threshold probabilities, supporting the practical application. The ROC of the predictive model was utilized to evaluate its significance in the modeling population, with an area under the ROC curve measuring 0.738 (Figure 2), indicating that the model has a fair predictive ability. Calibration curves were plotted for internal validation of the nomogram model, and the calibration curves closely approximated the ideal curves, indicating that the model exhibited strong discrimination and conformity (Figure 3). Decision curve analysis showed that the net clinical benefit for patients of using this model to screen for cognitive impairment was substantial when the probability value ranged from 1% to 86% (Figure 4).

## 4 Discussion

As PD advances, both motor symptoms deteriorate progressively, and non‐motor symptoms, including cognitive impairment, may emerge or exacerbate (Morris et al. 2024; Aarsland et al. 2021b). While the prominent motor symptoms of PD tend to receive the widest attention, the occurrence of non‐motor symptoms, such as cognitive impairment, is not uncommon, and the consequences are relatively serious, which needs to be given adequate attention (Liu et al. 2015). The H–Y classification, a common method for evaluating the severity of motor symptoms in PD patients, classifies the condition into Grades 0–5, with Grade 0 indicating normal function and Grade 5 representing the most severe stage. Previous multicenter studies (Kwon et al. 2022; Hosseini et al. 2023) demonstrated that the severity of motor symptoms and the stage of disease progression in PD patients are associated with the development of cognitive impairment and that the risk of cognitive impairment in patients increases with higher H–Y classification stages, the aggravation of motor symptoms (Tan et al. 2018). Some studies have confirmed that, in addition to the significant aggravation of motor symptoms, patients with moderate and advanced PD (H–Y classification 3–5) usually develop motor complications as well as non‐motor symptoms such as cognitive impairment, including executive dysfunction, memory loss, and decreased visuospatial ability, which seriously affects their daily life and self‐care ability (Siciliano et al. 2017). The study revealed that patients in the PD‐CI group exhibited a higher H–Y classification compared to the PD‐NC group, with a statistically significant difference (*p *< 0.05). The H–Y classification is a well‐established tool for assessing the severity of motor symptoms in PD patients and serves as a useful benchmark for tracking cognitive function changes in these individuals. The association between advanced H–Y stages and cognitive impairment aligns with Braak's theory of caudorostral neurodegeneration. As PD progresses to H–Y Stages 3–5, extranigral lesions in the locus coeruleus and limbic regions may concurrently drive motor deterioration and executive dysfunction, positioning H–Y staging as a surrogate marker for widespread neurodegeneration (Fontoura 2009). Therefore, higher H–Y stages in PD‐CI patients highlight its utility in tracking disease progression and associated cognitive decline.

Anxiety, a common non‐motor symptom in PD patients, profoundly affects their psychological health and overall quality of life. Braak's theory posits that the initial degeneration of the blueprint in PD patients may precede the dysfunction of the substantia nigra–striatum, the primary source of norepinephrine in the brain, which is crucial for regulating cerebral cortex activity; furthermore, the aberrant functioning of noradrenergic neurons has been linked to anxiety symptoms in these patients (C. Zhou et al. 2021; Braak et al. 2003). Research indicates that anxiety levels in PD patients may correlate with cognitive deficits, that functional connectivity between the left inferior frontal gyrus and the left amygdala is heightened in PD patients experiencing anxiety, and that the intensity of this functional connectivity positively correlates with the HAMA score, implying that the severity of anxiety symptoms may be associated with particular modifications in brain functional connectivity (Babaev et al. 2018; Carey et al. 2021; Vriend et al. 2016). Elevated HAMA scores align with findings of disrupted noradrenergic pathways in PD‐associated anxiety comorbidity, suggesting shared neurobiological substrates between anxiety and cognitive impairment (Qian et al. 2018). The results of this study indicated that patients in the PD‐CI group exhibited elevated HAMA scores compared to those in the PD‐NC group, and the difference between the two groups was statistically significant (*p *< 0.05). In the clinical management of PD patients, the HAMA score offers healthcare practitioners insights into the severity of patients' anxiety symptoms, aiding in therapy guidance and intervention formulation.

Hcy is a nonessential α‐amino acid intermediary in methionine (Met) metabolism. The metabolic pathway of Hcy encompasses remethylation, transsulfuration, and the synthesis of homocysteine lactone (HTL) (Kumar et al. 2017; Carboni 2022). The remethylation process transforms Hcy into Met through the involvement of vitamin B12 and 5‐methyltetrahydrofolate (5‐MTHF). The transsulfuration route transforms Hcy into cysteine and α‐ketobutyrate with the aid of vitamin B6. The synthesis of HTL entails the enzyme MARS, maybe through covalent alteration of the protein's lysine residues. Epidemiological investigations and clinical studies have demonstrated that the Hcy levels in PD patients are significantly elevated compared to healthy controls, indicating that Hcy may be a significant risk factor for PD (L. Zhou 2024; Padovani et al. 2016). Numerous studies suggest a correlation between Hcy levels and cognitive impairment in PD patients. A meta‐analysis indicated that PD patients with cognitive dysfunction exhibited elevated Hcy levels and diminished folate and vitamin B12 levels, implying a correlation between Hcy levels and cognitive impairment in this population (Ouyang et al. 2024). A case–control study pointed out that elevated Hcy levels were a risk factor for cognitive decline in PD. However, it did not establish a correlation between polymorphisms in genes associated with Hcy metabolism and cognitive impairment in PD (Periñán et al. 2023). A separate study investigated the relationship between serum UA, Hcy, and cystatin C levels and motor symptoms and cognitive function in PD patients (J. Li et al. 2020). The findings indicate that PD patients frequently experience shortages in vitamins, including folic acid, vitamin B6, and B12, attributable to lifestyle modifications and prolonged L‐dopa treatment, which may elevate blood levels of Hcy, resulting in hyperhomocysteinemia. Elevated Hcy levels may facilitate the progression of PD and the exacerbation of clinical symptoms by various mechanisms, including the induction of neuronal apoptosis, oxidative stress, mitochondrial malfunction, and DNA damage, particularly in cognitive decline (Mattson and Shea 2003; Mei et al. 2020). Moreover, elevated Hcy may further aggravate the immune‐inflammatory process in PD by stimulating immune responses and enhancing inflammatory reactions. The results of this study indicated that patients in the PD‐CI group exhibited elevated Hcy levels compared to those in the PD‐NC group, with the difference between the two groups being statistically significant (*p *< 0.05). While our data support Hcy as a risk factor, clinical trials testing folate supplementation for PD‐CI remain limited. Consequently, monitoring and lowering Hcy levels in PD patients may aid in delaying and managing the progression of cognitive impairment in these individuals. Supplementation with folic acid, vitamin B12, and vitamin B6 may lower Hcy levels, thereby decelerating cognitive decline in PD patients and enhancing prognosis and quality of life, which is crucial for the treatment and management of PD patients.

Folic acid is a crucial B vitamin for the human body, essential for preserving brain health; its lack may result in neurological damage and cognitive decline (Dong and Wu 2020). In PD patients, a notable correlation exists between folic acid levels and cognitive function, suggesting that folic acid may positively influence cognitive impairment in PD. Studies indicate that folic acid levels correlate with cognitive function in PD patients, and diminished folic acid levels may be related to cognitive function decline (Wei et al. 2016; Zhao et al. 2021). The investigation of the correlation among plasma concentrations of Hcy, 25‐hydroxyvitamin D (25‐(OH)‐D), and folic acid with cognitive function in elderly patients with PD revealed that Hcy levels were elevated in PD patients compared to controls, although 25‐(OH)‐D and folic acid levels were diminished relative to controls. In addition, as the clinical stage of the disease progressed, Hcy levels rose, whereas 25‐(OH)‐D and folic acid levels diminished. The results of this study indicated that patients in the PD‐CI group had lower folic acid levels compared to those in the PD‐NC group, with a statistically significant difference (*p *< 0.05). Folic acid is an independent risk factor for cognitive impairment in PD patients. Folic acid has antioxidant properties that can reduce levels of oxidative stress, which is considered one of the important triggers for neurodegenerative diseases (Liepelt‐Scarfone et al. 2021). In PD, supplementation of folic acid can alleviate neuroinflammatory responses and improve neurological function, further supporting the potential application of folic acid in the treatment of PD. Although research on the relationship between folic acid and cognitive impairment in PD is gradually increasing, more clinical trials are needed to verify the effectiveness and reliability of folic acid as a biomarker. Future research could focus on whether folic acid supplementation can improve cognitive function in PD patients and whether its mechanisms are related to neuroprotective effects.

UA, generated during purine metabolism, serves as a significant endogenous antioxidant, contributing to over fifty percent of the plasma's antioxidant capacity. It exerts neuroprotective effects through antagonism of the adenosine A2A receptor and its antioxidant activity. In PD patients, UA is associated with the severity of motor symptoms and the rapid progression of non‐motor symptoms, such as cognitive impairment. Cognitive functioning is likely superior in PD patients with elevated UA levels (Hemmati‐Dinarvand et al. 2019; Kachroo and Schwarzschild 2014). The prevailing consensus among scholars is that UA functions as a powerful antioxidant. They believe that a decrease in UA levels in PD patients increases oxidative stress and diminishes antioxidant capacity. This may result in the degeneration of dopaminergic neurons, potentially triggering or exacerbating both motor and non‐motor symptoms in PD patients (Grażyńska et al. 2021; Z. Yu et al. 2017; Wen et al. 2017; Schlesinger and Schlesinger 2008). Nevertheless, some studies have questioned the hypothesis that UA acts as an antioxidant. They propose that low levels of UA may be a consequence rather than a causative factor of PD. This viewpoint indicates that PD may result in reduced UA levels due to metabolic alterations associated with the disease or other influences. Another suggestion is that diminished levels of UA may function as a biomarker for other associated mechanisms in PD. This implies that low levels of UA may not be the direct cause of PD, but they might indicate the presence or severity of other underlying processes. A study by González‐Aramburu et al. (2014) involving 343 PD patients in Northern Spain revealed that, after adjusting for age, disease duration, cardiovascular disease, and other risk factors, there was no significant difference in serum UA levels between patients who developed PD dementia and those with normal cognitive function. The results of this study showed that patients in the PD‐CI group have lower UA levels than those in the PD‐NC group, with a statistically significant difference (*p *< 0.05). UA serves as an independent risk factor for cognitive impairment in PD patients. Therefore, monitoring UA levels may provide important information for the assessment of cognitive function in patients with PD. Although the potential of UA as a biomarker has been widely studied, more clinical research is needed to verify the specific role of UA and its mechanisms in cognitive impairment associated with PD (Wan et al. 2022). Future studies should focus on how to improve cognitive function in PD patients by regulating UA levels and exploring the interactions between UA and other biomarkers to provide more effective intervention strategies for clinical practice.

## 5 Conclusion and Prospect

Cognitive function in PD patients is affected by H–Y classification, HAMA scores, and levels of Hcy, UA, and folic acid. The constructed prediction model exhibits strong distinction and calibration as a reference for the early clinical detection and intervention of cognitive impairment in PD patients. A substantial proportion of PD patients exhibit cognitive impairment, which can be more accurately forecasted through the establishment of a risk prediction model. This model serves as a foundation for developing a comprehensive treatment plan, significantly contributing to the delay of cognitive decline and the enhancement of the quality of life for these patients.

This study possesses the subsequent limitations: (1) Inaccurate cognitive assessment: In 2012, the International Parkinson's Disease and Movement Disorders Society stipulated in its recommended diagnostic criteria for cognitive function in PD patients that the diagnosis should rely on a minimum of two test scores that fall below the mean of the normal control group by 1–2 standard deviations, alongside a restricted selection of neuropsychological assessments (Litvan et al. 2012). This study assessed the cognitive performance of PD patients using the MoCA score, a thorough evaluation of cognitive abilities; however, it has several limitations. Our reliance on MoCA alone (Level I criteria) may lack the sensitivity of comprehensive neuropsychological testing. MoCA is not sensitive enough for executive function and memory assessment and may underestimate subtype‐specific cognitive impairment (F. Li et al. 2019). Future research may include regional cognitive status assessment instruments, like the Hopkins Verbal Learning Test‐Revised (HVLT‐R), the Semantic Fluency Test (SF), and the Letter‐Number Sequencing Test (LNS), to improve diagnostic accuracy. (2) While the model showed net benefit across a wide probability threshold (1%–86%), this broad range may reflect cohort homogeneity. External validation in diverse populations is needed to confirm generalizability. (3) This study is a retrospective single‐center study with a constrained sample size, potentially resulting in biased outcomes, so prospective studies with larger sample sizes are necessary to corroborate the clinical applicability of the model further.

## Author Contributions

**Li Li**: data curation, writing–original draft. **Shan Tang**: conceptualization. **Bin Hao**: methodology. **Xiaoqin Gao**: investigation. **Haiyan Liu**: visualization. **Bo Wang**: formal analysis. **Hui Qi**: writing–review and editing, supervision.

## Ethics Statement

This retrospective study was approved by the Ethics Committee of the First Hospital of Shanxi Medical University (Ethics number: NO.KYLL‐2024‐260) and was conducted in accordance with the 1964 Helsinki Declaration and its later amendments or comparable ethical standards. Informed consent was waived by our Institutional Review Board because of the retrospective nature of our study.

## Conflicts of Interest

The authors declare no conflicts of interest.

### Peer Review

The peer review history for this article is available at https://publons.com/publon/10.1002/brb3.70423

## Data Availability

The datasets used and/or analyzed during the current study are available from the corresponding author upon reasonable request.
